# Whole-Genome Sequences of Four Strains Closely Related to Members of the *Mycobacterium chelonae* Group, Isolated from Biofilms in a Drinking Water Distribution System Simulator

**DOI:** 10.1128/genomeA.01539-15

**Published:** 2016-01-21

**Authors:** Vicente Gomez-Alvarez, Randy P. Revetta

**Affiliations:** U.S. Environmental Protection Agency, Office of Research and Development, Cincinnati, Ohio, USA

## Abstract

We report here the draft genome sequences of four *Mycobacterium chelonae* strains from biofilms subjected to a “chlorine burn” in a chloraminated drinking water distribution system simulator. These opportunistic pathogens have been detected in hospital and municipal water distribution systems, in which biofilms have been recognized as an important factor for their persistence.

## GENOME ANNOUNCEMENT

Despite the use of disinfectants in drinking water distribution systems (DWDS) to mitigate the presence of pathogens, a diverse and complex microbial community has been shown to inhabit DWDS (1). In chloraminated DWDS, the microbial community is often dominated by members of the *Mycobacterium chelonae-abscessus* complex (2). These rapidly growing nontuberculous mycobacteria (NTM) are opportunistic pathogens with the ability to form biofilms on the surface of DWDS (3). Furthermore, this complex has been implicated in invasive infections in immunocompromised hosts and is represented by *M. chelonae*, *M. abscessus*, *M. immunogenum*, *M. salmoniphilum*, *M. franklinii*, and *M. saopaulense* (4–10). Little information is available about the resilience of NTM in DWDS and the potential to cause public health problems (11, 12).

Strains from this study were isolated from biofilms obtained from a chloraminated DWDS simulator (3). Samples were collected from two distinct operational schemes (Table 1). Colonies were recovered from R2A plates after 7 days at 27°C. DNA was extracted using the UltraClean DNA microbial isolation kit, according to the manufacturer’s instructions (Mo Bio Laboratories, Solana Beach, CA). Paired-end 125-bp libraries were prepared using the Nextera XT DNA library kit, followed by rapid mode sequencing on the HiSeq 2500 platform (Illumina, Inc., San Diego, CA). Prior to assembly, libraries were (i) cleaned from contaminants (adapters, phiX, artifacts, and human), (ii) error corrected via Tadpole, (iii) normalized to ≤100×, (iv) removed of low-coverage (<6×) reads, and (v) filtered to a minimum length read of 125 nucleotide (nt). The reads were processed using the software package BBMap version 35.34 (http://sourceforge.net/projects/bbmap) and *de novo* assembly with SPAdes version 3.5.0 (13). The final assembly attributes are listed in Table 1.

**TABLE 1:** Summary statistics of whole-genome assemblies

Strain	Source (surface)	Operational scheme^a^	Fold coverage (×)	No. of contigs	Contig *N*_50_	Assembly size (bp)	G + C content (%)	Accession no.
H002	Biofilm (Cu)	Standard I	74	60	192,652	5,421,590	64.00	LJYI00000000
H003	Biofilm (PVC)	Standard I	91	35	515,028	5,588,459	63.94	LJYL00000000
H072	Biofilm (Cu)	Standard II	79	54	210,836	5,449,380	64.00	LJYK00000000
H079	Biofilm (PVC)	Standard II	50	94	170,224	5,726,565	63.90	LJYO00000000

a Standard I, stable chloramine residual; Standard II, stable chloramine residual after a “chlorine-burn.”

The average nucleotide identity (ANI), a similarity index between two genomes (14), grouped the four strains into two subclusters (subclusters 2a and 2b). The genome similarity between the two subclusters is 97.245% to 97.553%, with an ANI within all strains of 99.960%. The proposed cutoff for species is 95% to 96% (15). Coincidentally, strains of subclusters 2a and 2b were obtained from biofilms attached to polyvinyl chloride (PVC) and copper (Cu) surfaces, respectively. The isolates share an overall 95.086% ANI with *M. chelonae* ATCC 35752 (6) and 83.008% to 83.275% ANI with *M. abscessus* ATCC 19977 (7) and *M. immunogenum* SMUC14 (8), respectively. ANI calculations were performed using the online calculator available from EzGenome (http://www.ezbiocloud.net/ezgenome/ani). Furthermore, a comparative analysis of the *rpoB*, *recA*, and *sodA* genes revealed <95.04% ± 0.05% sequence homology with representatives of the *M. chelonae-M. abscessus* complex (6–10). Genomic comparison confirmed that these isolates belong to the *M. chelonae* group but may constitute a different subspecies.

Genome assemblies were annotated with Prokka version 1.10 (16), available as an application in Illumina BaseSpace Labs. The genome sequence of strain H002 contains 5,378 genes, 5,333 coding sequences (CDSs), 3 rRNAs, and 41 tRNAs; strain H003 contains 5,635 genes, 5,551 CDSs, 3 rRNAs, and 81 tRNAs; strain H072 contains 5,410 genes, 5,362 CDSs, 3 rRNAs, and 45 tRNAs; and strain H079 contains 5,824 genes, 5,362 CDSs, 3 rRNAs, and 76 tRNAs.

### Nucleotide sequence accession numbers.

The whole-genome shotgun project has been deposited in DDBJ/ENA/GenBank under the accession numbers listed in Table 1. The versions described in this paper are the first versions.
